# Medicine and the Liberal Spirit

**Published:** 1911-06-17

**Authors:** 


### Medicine and the Liberal Spirit.

An amount of lay criticism rather unusual at this busy season has been provoked by the recent disciplinary action of the Medical Council in removing from the Register medical men who had in various ways covered unqualified persons' treatment of patients. It never takes much to make the outsider scent expert official prejudice, and unfortunately the phrase "medical etiquette" is one which especially stinks in his nostrils. Added to this the medically quite uninformed person—a description which fits nine-tenths of the leaders of public opinion—though necessarily an incompetent judge, is pathetically and dangerously unaware of his condition. He does not know how one thing alone—the *vis medicatrix naturæ*—vitiates what he regards as an infallible test—namely, the testimony of the patient. He has no faint inkling of the difficulties to be met with, as regards medical treatment, in the category of cause and effect; while here, unfortunately, too many medical men are in the same boat with him. "There is no drink or stink," says a plain-spoken author, "which has not been tried in phthisis"—which, too, he might have added, has not with easy confidence been said to do good. Then, too, the journalist critic, probably something of a classical scholar, apparently conceives of present-day medicine as still in the Greek or Roman, at any rate in the mediæval age.

The sober truth is that it may be laid down as all but axiomatic that no one lacking medical training can find out anything fresh about European diseases; as far as this, now, the force of nature cannot go: as well expect it to as an agricultural labourer to fill a chair of botany, or a biblewoman a place on an exegetical committee. If, then, the medical profession fails in liberality of opinion, such failing has not appeared in any recent public act. To establish the existence of such failing deeper and more esoteric arguments would be necessary. It is a fair charge, perhaps, that originality is undervalued, but then that is what generally happens to the possessors of this gallant quality. In particular do some of our leaders hate anything in the nature of a theory, and in support of their attitude contrast the old crude hypotheses and the errors they led into, with the results of the experimental method of Koch. But the extraordinary speculations of such a man as Gall, for instance, were the product of a mind that had no perception of the requirements of the scientific method. He had not been taught the need of verification, which may always be trusted to prune any luxuriance of fancy in an honest worker. And on the other hand the present tendency to reduce research to an affair of technique and of tabulating findings, is forcing such a jungle of facts—or probable facts—as is likely to overtax the strongest associative faculty. "Science is measurement," said Herder, and so in some part it is. But there exists a commonplace of logic to the effect that measurement can never be exact; and at the present time our experimental medical science bears out this proposition completely. Many of the theorems of causation attacked are far too complex to be solved in the present state of knowledge. Vary one of the numberless conditions of the experiment ever so slightly and the result alters surprisingly. Accordingly we have the common spectacle of careful crucial experiments contradicting each other; as, for instance (by his own account), in the work of a member of the recent Tuberculosis Commission. The rich variety of interpretative and critical faculty displayed in different minds adds more diversity still. In short, it is no news at this time of day that in pure induction Bacon gave the world a much less valuable *organon* than he thought. Only when the issue has been simplified by prevision, only when induction and deduction are used as Newton used them—as leaping pole and scaling ladder—can experiment yield decisive data.

Another thing to beware of is making too much of the examination test. There are certain hardly awarded degrees made essential to candidates for important appointments, and a very valuable safeguard against incompetence, no doubt, the possession of them is. To get into the running for posts of great clinical opportunity the aspirant must never diverge from a beaten path of study, and the requirement may be the reverse of congenial to a gifted mind. At the end of it young men are commonly exhorted to climb up on the shoulders of an elder by working at his particular line of study, a proceeding which may be useful enough as regards ordinary ability, but which in the case of real investigatory talent is the worst possible employment of those years of brisk brain which seldom last after forty. Again, it is a short step from the crying down of individuality to a more or less declared attitude of downright academic intolerance. All these are reasons why our young clinicians write useless textbooks instead of useful monographs, and why the unnecessary proposal has been made to constitute consultants into an altogether separate, higher branch of the profession—a scheme good comment upon which is Dr. Mackenzie's recent statement that certain kinds of heart disease can be studied by the general practitioner only. Indeed, although recent lay charges quite fall to the ground, the profession has everything to gain by cultivating a liberal spirit.

